# An empirical application of “broken windows” and related theories in healthcare: examining disorder, patient safety, staff outcomes, and collective efficacy in hospitals

**DOI:** 10.1186/s12913-020-05974-0

**Published:** 2020-12-04

**Authors:** Louise A. Ellis, Kate Churruca, Yvonne Tran, Janet C. Long, Chiara Pomare, Jeffrey Braithwaite

**Affiliations:** Centre for Healthcare Resilience and Implementation Science, Australian Institute of Health Innovation, Macquarie University, Sydney, Australia

**Keywords:** Broken windows theory, Disorder, Collective efficacy, Hospital, Safety, Culture

## Abstract

**Background:**

Broken windows theory (BWT) proposes that visible signs of crime, disorder and anti-social behaviour – however minor – lead to further levels of crime, disorder and anti-social behaviour. While we acknowledge divisive and controversial policy developments that were based on BWT, theories of neighbourhood disorder have recently been proposed to have utility in healthcare, emphasising the potential negative effects of disorder on staff and patients, as well as the potential role of collective efficacy in mediating its effects. The aim of this study was to empirically examine the relationship between disorder, collective efficacy and outcome measures in hospital settings. We additionally sought to develop and validate a survey instrument for assessing BWT in hospital settings.

**Methods:**

Cross-sectional survey of clinical and non-clinical staff from four major hospitals in Australia. The survey included the Disorder and Collective Efficacy Survey (DaCEs) (developed for the present study) and outcome measures: job satisfaction, burnout, and patient safety. Construct validity was evaluated by confirmatory factor analysis (CFA) and reliability was assessed by internal consistency. Structural equation modelling (SEM) was used to test a hypothesised model between disorder and patient safety and staff outcomes.

**Results:**

The present study found that both social and physical disorder were positively related to burnout, and negatively related to job satisfaction and patient safety. Further, we found support for the hypothesis that the relationship from social disorder to outcomes (burnout, job satisfaction, patient safety) was mediated by collective efficacy (social cohesion, willingness to intervene).

**Conclusions:**

As one of the first studies to empirically test theories of neighbourhood disorder in healthcare, we found that a positive, orderly, productive culture is likely to lead to wellbeing for staff and the delivery of safer care for patients.

**Supplementary Information:**

The online version contains supplementary material available at 10.1186/s12913-020-05974-0.

## Background

A long tradition exists in criminology and social-psychology research on the concept of neighbourhood *disorder* and in what ways disorder relates to anti-social behaviour and poor outcomes [1]**.** Interest in neighbourhood disorder is readily apparent in Broken Window Theory (BWT) [2], as well as in alternative perspectives of disorder involving shared expectation and cohesion—more broadly known as *collective efficacy* [3–5]—that are consistent with social disorganisation theory. The current study draws from these various theories and insights into neighbourhood disorder and applies them to hospital settings. At this point, we must make clear our intentions in applying neighbourhood disorder theories to healthcare. It is perilous to expect theories of neighbourhood disorder can be perfectly replicable in an organisational setting, nor do we consider that all elements of the theories are applicable to hospital settings (such as the concept of fear) [6]**.** We particularly reject the flawed ramifications of these theories that saw victimisation and blame attributed to individual neighbourhood members. However, here, we consider that concepts from neighbourhood studies may have considerable promise to shed new light on the relationships between the physical and social environments of hospitals on the one hand, and the health, wellbeing and behaviour of staff and patients, on the other [7]**.** We begin by reviewing the history and evolution of these theories before considering their application to healthcare.

### Broken windows: a theory of disorder in neighbourhoods

Broken windows theory (BWT), as a social-psychological theory of urban decline, was originally developed almost 40 years ago by Wilson and Kelling [2]. Proponents of this theory argue that both physical disorder (e.g., broken windows, graffiti, litter) and social disorder (e.g., vandalism, antisocial activities) provide important environmental cues to the kinds of negative actions that are normalised and tolerated in an area, fuelling further incivility and more serious crime. For example, signs of disorder can signal potential safety issues to residents of a neighbourhood, leading to their withdrawal from public spaces, and thereby a reduction in informal social control, further perpetuating the effects of disorder [2].

### Defining disorder

Although debates have occurred in the literature as to what counts as disorder, it has usually been defined as representing “minor violations of social norms” ([8] p4923). Some researchers have made a distinction between physical and social disorder, with physical disorder relating to the overall appearance of an area and social disorder directly involving people [9]. Thinking about disorder in this way, neighbourhoods with high levels of physical disorder were defined as: noisy, dirty, and run-down; buildings are in disrepair or abandoned; and vandalism and graffiti are common [10]. On the other hand, signs of social disorder in neighbourhoods may include the presence of people hanging out on the streets, drinking, or taking drugs [10]. Researchers highlight the importance of measuring perceptions of physical and social disorder as separate factors [9, 11] with recent studies finding differential impacts of the two types of disorder [12].

### Rethinking disorder: the role of collective efficacy

The BWT originally proposed by Wilson and Kelling [2] suggested a causal relationship with disorder leading to crime, which had a significant bearing upon subsequent controversial policy developments, such as ‘zero-tolerance policing’ [13] and ‘stop-and-frisk’ programs [14]. Under this approach, police pay attention to every facet of the law, including minor offences, such as public drinking and vandalism, with the aim of preventing more serious crimes from occurring [13]. The level of support these policing strategies have received has been surprising, given that BWT has not received a commensurate amount of study to date, and the research on crime that does exist is equivocal [12]. In particular, there has been an ongoing debate in the academic literature over whether BWT posits a direct or indirect relationship between disorder and crime. Most prominently, Sampson and Raudenbush [4] reconsidered the claims of BWT and argued instead that physical and social disorder were not generally causal antecedents to more serious crimes. Consistent with social disorganisation theory [3], Sampson and Raudenbush [4] suggested that *collective efficacy* has a significant influence on criminality in neighbourhoods. They defined collective efficacy as “social cohesion among neighbours combined with their willingness to intervene on behalf of the common good” ([5] p918). Empirical results supported their conceptual ideas in that the positive relationship between disorder and crime was mediated by collective efficacy [4].

Other lines of research have found a direct association between disorder and crime even when controlling for collective efficacy (e.g., [15]). For example, Plank et al. [16] studied disorder and collective efficacy in a school setting. They found a robust association between both disorder and violence (i.e., crime) while controlling for collective efficacy. They concluded that “fixing broken windows and attending to the physical appearance of the school cannot alone guarantee productive teaching and learning, but ignoring them greatly increases the chances of a troubling downward spiral” ([16] p244). In summary, the results are mixed as to the extent that there is direct effect of disorder on crime or other poor outcomes, but the evidence clearly suggests that there is at least an indirect effect. The key problem is what people do with this information. There is no justification for blaming individuals or demonising groups or neighbourhoods for their behaviour. We do not in any way condone seriously erroneous and consequential victimisation of people or groups as a result of the application of BWT. But we do think this is an area worthy of study.

### Applying broken windows theory to healthcare

Following recent interest in applying BWT to smaller, more circumscribed environments, such as workplaces [17, 18], researchers have started to consider the application of BWT to healthcare settings [7, 19, 20]. There are several well-studied trends in health services research that support this application. Theories and studies of increasing popularity include: the normalisation of deviance [21], behavioural modelling in hand hygiene [22], hospital workplace violence [23], and the association between staff’s safe work practices and their perceiving their work area as cluttered and disorderly [24].

**Fig. 1:**
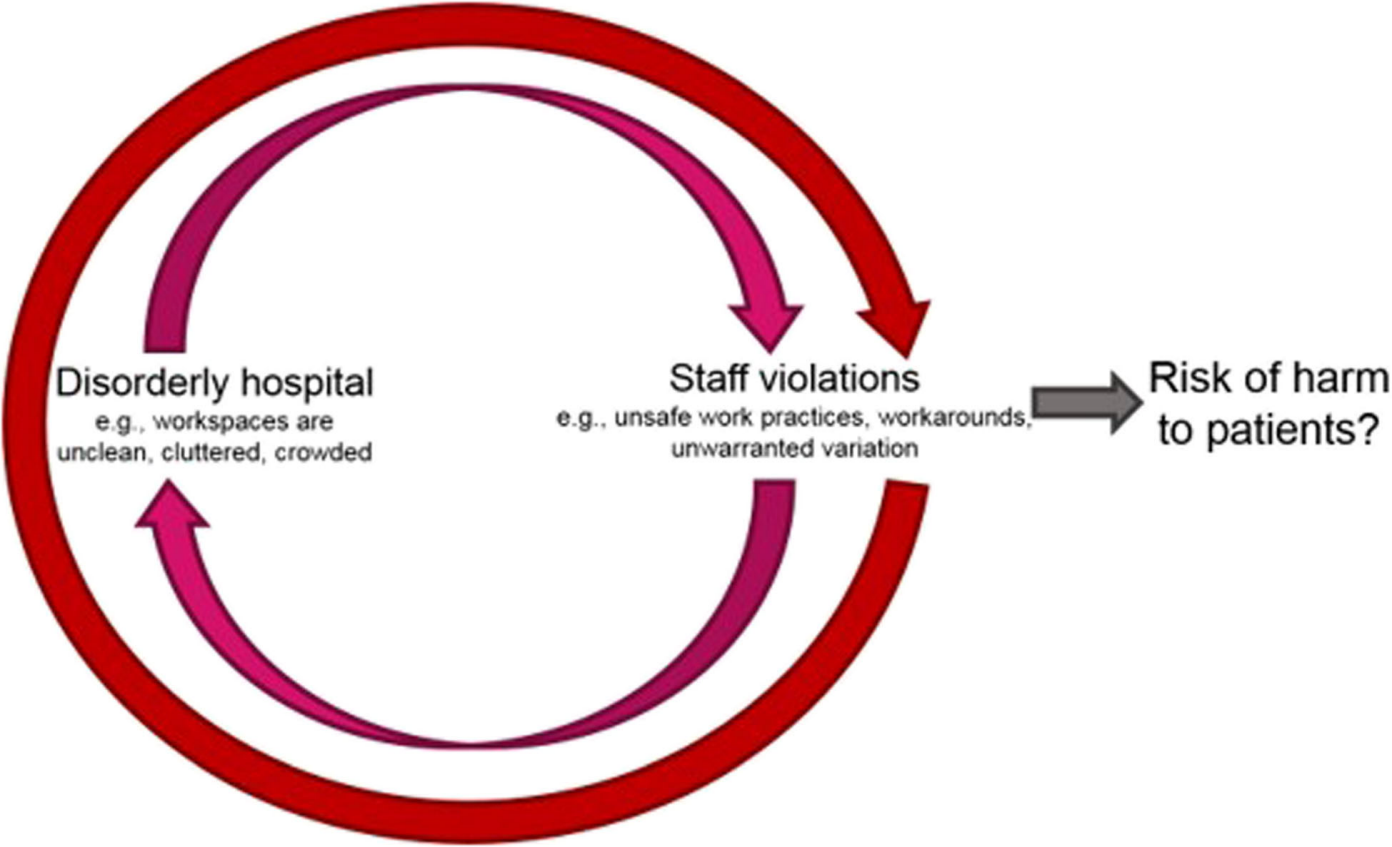
Proposed model of disorder in hospitals Source: Churruca, Ellis et al., 2018 [7]

Disorder in hospitals may include negative deviations, trade-offs or workarounds that manifest continuously in complex, dynamic and time-pressured environments, which can contribute to poor staff outcomes [25–27]. While trade-offs and workarounds occur in every setting, and they may have many benefits including signalling productive flexibility and staff capacity for manoeuvring, they can also represent risk in healthcare. For example, some researchers have shown that small deviations such as violating recommended processes for use of local anaesthesia can be detrimental, potentially even leading to death [28]. In line with BWT logic, there is evidence to suggest that the physical hospital environment influences the health and wellbeing of staff and patients [29]. Similarly, evidence shows that social disorder (e.g., bullying, violence) can influence staff in healthcare organisations [23, 30]. All of these examples highlight the potential negative perpetuating effects of disorder in healthcare organisations and how disorder may detrimentally affect patients, such as through poor patient safety outcomes (see Fig. 1 [7]). Despite the elevated interest in BWT, we could find no empirical study of disorder in hospitals, nor any examination of the role of collective efficacy on staff outcomes or patient safety.

### Aims of the present study

**Fig. 2:**
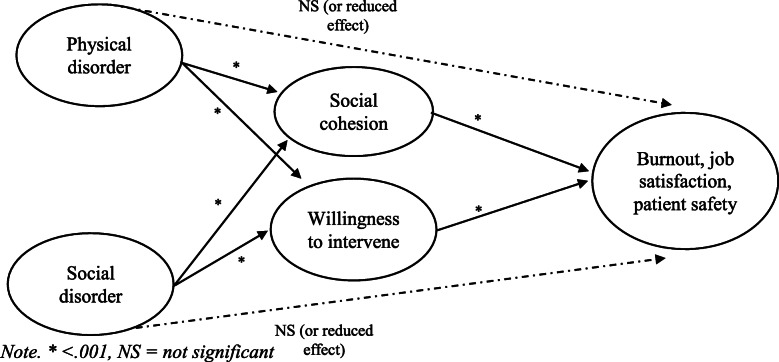
Hypothesised mediation model

The primary purpose of the present study is to empirically examine the relationship between hospital disorder and three key outcomes: staff burnout, staff job satisfaction, and patient safety. We also sought to address the contention in the literature regarding the role of collective efficacy (defined here as social cohesion among hospital staff and their willingness to intervene to address problems) between hospital disorder and outcomes. The first aim was to develop a short but valid and reliable survey instrument for measuring physical disorder, social disorder, social cohesion and willingness to intervene in hospital settings. Based on previous research, physical and social disorder were kept as separate constructs. We then sought to test the following three research questions:

1. Is there a significant association between hospital disorder (physical disorder, social disorder) and staff outcomes (burnout, job satisfaction)?
2. Is there a significant association between hospital disorder (physical disorder, social disorder) and patient safety?
3. What is the function of “collective efficacy” (social cohesion, willingness to intervene) in hospitals? Specifically, does staff collective efficacy mediate the relationship between disorder and outcomes? Figure 2 demonstrates the simplified hypothesised mediation model.

## Methods

### Participants and setting

The study employed a cross-sectional survey of staff from four major hospitals in Australia. All hospital sites were public hospitals in metropolitan areas with over 200 beds. The sites were selected based on the similarity in the types of services offered (e.g., emergency department, intensive care, surgical, medical, geriatric care) and that they were located within areas of varying relative socio-economic disadvantage [31]. All hospital staff were invited to participate in the study through an invitation sent to their work email address. The email included a link to an online version of the survey via *Qualtrics* [32].

### Survey development

The Disorder and Collective Efficacy survey (DaCEs) for hospital staff was developed for the present study based on an extensive review of the BWT literature. An initial pool of items was formed to assess the hypothesised constructs of the DaCEs: Physical disorder (19 items), social disorder (13 items), and collective efficacy, represented by social cohesion (12 items) and willingness to intervene (10 items). Some of the items were adapted from existing scales [16, 24, 33–35], and others were purpose-developed by the research team (see Supplementary File 1). Items were modified to make them relevant to a hospital context. All items were answered on a five-point Likert scale (1 = strongly disagree to 5 = strongly agree). A panel of experts in healthcare (*n* = 10; hospital staff and researchers) reviewed and provided feedback on the wording of items mapping onto each of the hypothesised constructs and checked for possible misinterpretations of questions, instructions and response format. Minor adjustments were made to the initial item pool (see Supplementary File 1). The aim was then to refine the item pool to produce a survey that would be short enough to be completed by busy hospital workers, but which has satisfactory psychometric properties.

#### Staff outcomes

The survey included existing validated scales to measure staff burnout and job satisfaction. Burnout was measured through a 10-item version of the Maslach Burnout Inventory (MBI) [36–38]. Two subscales of burnout—emotional exhaustion and depersonalisation—were used for the current survey as the third subscale, personal accomplishment, was deemed less relevant to nonclinical staff. Burnout items were answered on a seven-point Likert scale (1 = strongly disagree to 7 = strongly agree). The job satisfaction section of the Job Diagnostic Survey (5 items) was selected to capture individual’s feelings about their job [39]. Job satisfaction items were answered on a five-point Likert scale (1 = strongly disagree to 5 = strongly agree).

#### Patient safety

An item taken from the Hospital Survey of Patient Safety Culture (HSOPSC) was used as an indicator of patient safety [40]. This item is an outcome measure for patient safety that asks staff to provide an overall patient safety grade for their hospital (1 = excellent to 5 = failing).

### Data analysis

Participants missing more than 10% of survey data were excluded. Remaining missing values were imputed using the Expectation Maximisation (EM) Algorithm within SPSS, version 25 [41]. Some items were then reversed coded so that higher item-response scores indicated a greater extent of job satisfaction, burnout, disorder, willingness to intervene, and patient safety (See Supplementary File 1 for individual recoded items). Frequency distributions were calculated to test whether items violated the assumption of univariate normality (i.e., skewness index ≥3, kurtosis index ≥10). As a number of the items were skewed (i.e., skewness index ≥3), the chi-square significance value was corrected for bias using the Bollen-Stine bootstrapping method [42] based on 1000 bootstrapped samples.

Items were evaluated psychometrically via confirmatory factor analysis (CFA), using a two-stage process. First, to refine the initial item pool, four one-factor congeneric models (of physical disorder, social disorder, social cohesion and willingness to intervene items) were run using AMOS, version 25 [43]. Here, our analytic plan involved removing one item at a time from each model using the following strategy: (i) removing items with the lowest factor loadings while maintaining the theoretical content and meaning of the proposed construct; (ii) removing items as long as each construct contained at least four observed variables; and (iii) items were removed as long as the resulting model demonstrated an improved model fit [44, 45]. Differences in model fit were assessed using the chi-square difference test [46]. Second, two two-factor models were used to assess the factor structure of items related to disorder (i.e., physical disorder, social disorder) and collective efficacy (i.e., social cohesion, willingness to intervene) using the reduced item sets. Each item was loaded on the one factor it purported to represent. Further item refinement was undertaken as required through inspection of factor loadings, standardised residuals and modification indices to reduce each scale to three or four items. Goodness-of-fit was assessed using the Tucker Lewis Index (TLI), Comparative Fit Index (CFI) and Root Mean Square Error of Approximation (RMSEAs), and chi-square, with significance value supplemented by the Bollen-Stine bootstrap test. The TLI and CFI yield values ranging from zero to 1.00, with values greater than .90 and .95 being indicative of acceptable and excellent fit to the data [47]. For RMSEAs, values less than .05 indicate good fit, and values as high as .08 represent reasonable errors of approximation in the population [48]. For the Bollen-Stine test, non-significant values indicate that the proposed model is correct. Reliability of each of the subscales was assessed through Cronbach’s alpha (using SPSS, version 25) and composite reliability (using AMOS, version 25).

The hypothesised mediation model (Fig. 2) was assessed using structural equation modelling (SEM) in AMOS, version 25 [43]. First, we tested the direct effects from disorder (physical and social) to each outcome (burnout, job satisfaction, patient safety), followed by the indirect effect from disorder to outcomes, through collective efficacy (social cohesion, willingness to intervene). A parametric bootstrapping approach was used to test mediation. Under the bootstrapping approach, indirect effects are of interest and based on bootstrapped standard errors (with 1000 draws) [49, 50]. Model fit was evaluated using CFI, TLI, RMSEA, and chi-square.

## Results

### Descriptive statistics, distribution, reliability and confirmatory factor analysis

Participants were 415 staff from four hospitals in Australia. Once participants with more than 10% of survey data missing were excluded, the remaining sample was reduced to 340. Of the 340 participants, most were female (77.5%), worked as a nurse (34.2%), and had been working in the same hospital for three or more years (76.1%). The characteristics of the survey respondents are presented in Table 1.

**Table 1:** Characteristics of survey respondents (*n* = 340)

	***n***	***%***
Sex		
Male	75	22.5
Female	259	77.5
Age		
18–24 years	9	2.6
25–34 years	83	24.4
35–44 years	76	22.4
45–54 years	91	26.8
> 55 years	81	23.8
Years at hospital		
< 1 year	40	12.0
1–2 years	40	12.0
3–5 years	71	21.3
6–10 years	74	22.2
> 11 years	109	32.6
Role		
Administration/Clerical	50	14.7
Allied health professional	48	14.2
Management	26	7.7
Physician/Medical officer	62	18.3
Registered or enrolled nurse	116	34.2
Other (e.g., volunteer, pharmacist, scientist)	37	10.9

Note. Columns may not equal total N due to missing demographic responses

Descriptive statistics and data pertaining to assumptions of normality for all items are presented in Supplementary File 1. The vast majority of the social disorder, social cohesion and willingness to intervene items demonstrated a skewness index greater than three, while only three items demonstrated a kurtosis index greater than 10 (SD7, SD10, SC6). As a result, Bollen-Stine bootstrapping was conducted in order to improve accuracy when assessing parameter estimates and fit indices.

To refine the initial item pool, first four one-factor congeneric models were run for items designed to measure physical disorder, social disorder, social cohesion and willingness to intervene. Based on an examination of modification indices and standardised factor loadings, items were removed one at a time, until the four strongest items remained. As shown in Table 2, the reduced four-item constructs demonstrated much improved model fit statistics relative to the full models with all items. Chi-squared difference tests for all four constructs were significant, indicating that the reduced item constructs were significantly better models. The results of the chi-squared difference tests were: Physical disorder, (χ^2^ difference = 139, df = 18, *p* < .001), social disorder (χ^2^ difference = 680, df = 63, *p* < .001), social cohesion (χ^2^ difference = 302, df = 52, *p* < .001), and willingness to intervene (χ^2^ difference = 243, df = 33, *p* < .001).

**Table 2:** Model fit for the one-factor congeneric models

Construct	χ^2^	df	TLI	CFI	RMSEA
Physical disorder					
All items (8 items)	149.58	20	.80	.86	.14
Reduced items (4 items)	10.49	2	.96	.99	.11
Social disorder					
All items (13 items)	691.85	65	.66	.72	.17
Reduced items (4 items)	12.15	2	.96	.99	.12
Social cohesion					
All items (12 items)	303.69	54	.88	.90	.12
Reduced items (4 items)	1.75	2	1.00	1.00	.00
Willingness to intervene					
All items (10 items)	244.83	35	.81	.85	.13
Reduced items (4 items)	1.54	2	1.00	1.00	.00

Two two-factor models of disorder (physical disorder, social disorder) and collective efficacy (social cohesion, willingness to intervene) were then tested through CFA each using eight of their respective items. Each item was loaded on the one factor it purported to represent. Where required, further item refinement was undertaken through inspection of factor loadings, standardised residuals and modification indices. The two-factor model of disorder, including four physical disorder items and four social disorder items produced an adequate fit to the data, χ^2^ (19) = 54.06, TLI = .96, CFI = .97, RMSEA = .08, though the Bollen-Stine bootstrap was significant (*p* = .005). Inspection of the standardised factor loadings for items PD3 and SD3 suggested that their removal may improve model fit. The removal of these two items resulted in an improved model fit, χ2 (8) = 18.28, TLI = .979, CFI = .989, RMSEA = .062, and the Bollen-Stine bootstrap (*p* = .057). The standardised factor loadings for the six items remaining ranged from .71 to .90. The correlation between physical disorder and social disorder was low, but significant (*r* = .17, *p* = .007). Next, a two-factor model of collective efficacy consisting of four social cohesion items and four willingness to intervene items were tested. This model produced an excellent fit to the data, χ2 (19) = 25.36, TLI = .99, CFI = 1.00, RMSEA = .06, and the Bollen-Stine bootstrap was not significant (*p* = .458). The standardised factor loadings for the six items ranged from .68 to .90, and the correlation between social cohesion and willingness to intervene was strong, *r* = .69, *p* < .001. The retained items from the two-factor models are presented in Table 3, along with their factor loadings. Cronbach’s alpha and composite reliability for the final items is also shown in Table 3, demonstrating that all four scales demonstrated acceptable levels of reliability.

**Table 3:** CFA results for reduced two factor models of disorder and collective efficacy

Construct	Item	Factor loadings	Coefficient alpha	Composite reliability
**Model 1: Disorder**				
Physical disorder	PD1	.90	.84	.80
	PD2	.71		
	PD7	.81		
Social disorder	SD4	.84	.86	.86
	SD5	.82		
	SD6	.80		
**Model 2: Collective efficacy**				
Social cohesion	SC1	.87	.91	.90
	SC2	.88		
	SC3	.90		
	SC5	.81		
Willingness to intervene	WI6	.68	.85	.83
	WI8	.82		
	WI9	.83		
	WI10	.77		

### Research question 1: is there a significant association between hospital disorder and staff outcomes?

In order to examine the relationship between hospital disorder and staff outcomes, four separate models were run (i.e., models were run separately for physical disorder and social disorder, each with burnout and job satisfaction as dependent variables). Findings are presented in Supplementary File 2. The results showed that physical disorder was significantly associated with higher burnout (β = .26, *p* < .001) and lower job satisfaction (β = −.40, *p* < .001). Similarly, social disorder was significantly associated with higher burnout (β = .23, *p* < .001) and lower job satisfaction (β = −.54, *p* < .001).

### Research question 2: is there a significant association between hospital disorder and patient safety?

Two separate models were run for physical disorder and social disorder (Supplementary File 2). Physical disorder was significantly associated with lower patient safety scores (β = −.15, *p* = .008). Likewise, a greater extent of social disorder was significantly associated with lower levels of patient safety (β = −.26, *p* < .001).

### Research question 3: does staff collective efficacy mediate the relationship between disorder and outcomes?

**Fig. 3:**
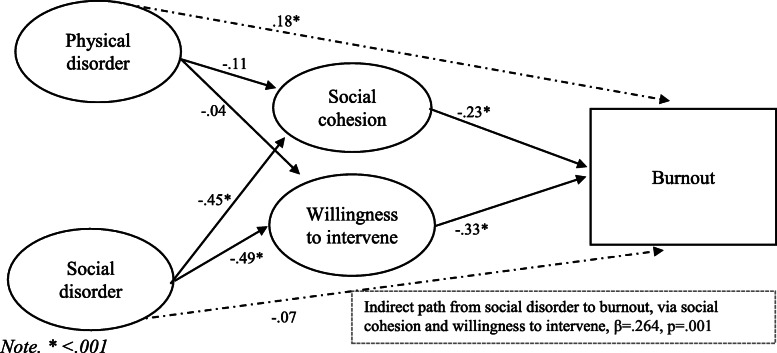
Model of disorder and burnout, mediated by collective efficacy

We then tested three separate mediation models for each outcome measure where the relationship between disorder and outcomes was mediated by collective efficacy via bootstrapping. For burnout, the model fit the data well, χ2 (81) = 142.75, TLI = .97, CFI = .98, RMSEA = .05. The findings presented in Fig. 3 show that there were significant negative paths from: social disorder to social cohesion (β = −.45, *p* = .003); social disorder to willingness to intervene (β = −.49, *p* = .002); social cohesion to burnout (β = −.23, *p* = .022); and willingness to intervene to burnout (β = −.33, *p* = .004). However, the paths from physical disorder to social cohesion (β = −.11, *p* = .077) and from physical disorder to willingness to intervene (β = −.04, *p* = .466) were not significant. Alongside these parameters, there was a significant direct effect from physical disorder to burnout (β = .18, *p* = .001), but not from social disorder to burnout (β = −.07, *p* = .351). Importantly, bootstrapped analyses for indirect effects indicated a significant indirect path from social disorder to burnout via social cohesion and willingness to intervene (β = .26, *p* = .001). However, the indirect path from physical disorder to burnout was not significant (β = .04, *p* = .205).

**Fig. 4:**
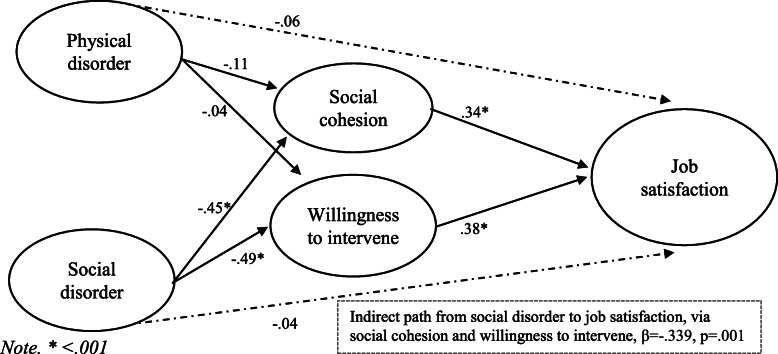
Model of disorder and job satisfaction, mediated by collective efficacy

For job satisfaction, the model provided an adequate fit to the data, χ2 (125) = 274.69, TLI = .95, CFI = .96, RMSEA = .06 (Fig. 4). The findings show that there was a significant path from social cohesion to job satisfaction (β = .34, *p* = .002) and from willingness to intervene to job satisfaction (β = .38, *p* = .001). The direct effects from physical disorder to job satisfaction (β = −.06, *p* = .233) and from social disorder to job satisfaction (β = −.04, *p* = .575) were not significant. Bootstrapped analyses for indirect effects indicated a significant indirect path from social disorder to job satisfaction via social cohesion and willingness to intervene (β = −.34, *p* = .001). However, the indirect path from physical disorder to burnout was not significant (β = −.05, *p* = .171).

**Fig. 5:**
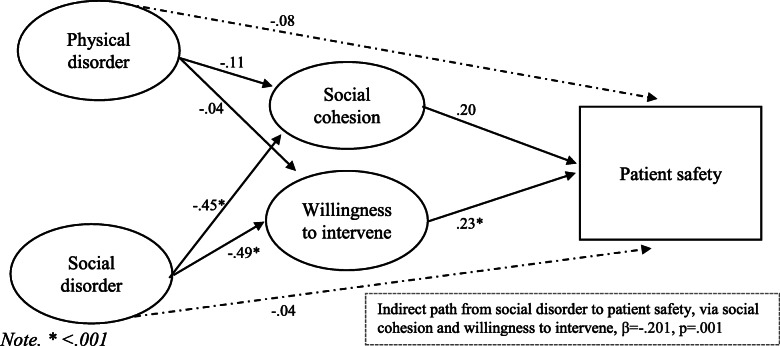
Model of disorder and patient safety, mediated by collective efficacy

For patient safety, the model fit provided a satisfactory fit to the data, χ2 (81) = 171.26, TLI = .96, CFI = .97, RMSEA = .06. The findings are presented in Fig. 5 and show that there was a significant path from willingness to intervene to patient safety (β = .23, *p* = .041). The path from social cohesion to patient safety just failed to reach significance (β = .20, *p* = .057). The direct effects from physical disorder to patient safety (β = −.08, *p* = .155) and from social disorder to patient safety (β = −.04, *p* = .612) were not significant. The indirect effects indicated a significant indirect path from social disorder to patient safety via social cohesion and willingness to intervene (β = −.20, *p* = .001). However, the indirect path from physical disorder to burnout was not significant (β = −.03, *p* = .174).

## Discussion

BWT and related theories of neighbourhood disorder were used here as a novel way of studying the influence of hospital environment on staff outcomes and patient safety. In this study, we developed and validated a survey instrument of disorder and collective efficacy for hospital staff—the DaCEs. In response to our research questions, we found that both social and physical disorder were positively related to burnout and negatively related to job satisfaction and patient safety. This indicated that the greater the perceived disorder in hospitals the higher the burnout and lower job satisfaction in hospital staff, and lower ratings of patient safety. Although neighbourhood disorder theories are not perfectly applicable to a hospital setting, our findings are broadly analogous with previous neighbourhood research and suggest that while attending to the physical appearance of the hospital cannot alone guarantee better staff and patient outcomes, ignoring them can significantly increase the chances of poorer outcomes. The present study also found support for the contention that collective efficacy mediated the relationship between social disorder and outcomes (burnout, job satisfaction, patient safety), but not for physical disorder.

This study is one of the first to empirically evaluate neighbourhood disorder theories in healthcare. Consistent with the original BWT, we found that perceptions of social and physical disorder were associated with potential safety issues [2], in this case, low patient safety ratings in hospitals. Past research on neighbourhood disorder supports the association between perceived neighbourhood disorder and poor mental health [51], corresponding with the present study’s findings that hospital disorder was associated with low job satisfaction and high burnout. These findings shed light on the potential relationship between culture and disorder in hospitals. We recognise that BWT has received considerable criticism over the years [1], particularly in response to controversial policy developments that were based on the BWT perspective. At this point, we must make clear that we do not advocate such policies, and find them abhorrent. However, we do contend that it seems likely that disorder is a marker for a poorer workplace culture compared to a workplace that is perceived as more orderly by hospital staff. This represents further converging evidence that having a productive, functional, more orderly culture is good for both staff and patients and not having a collective, efficacious, productive, collaborative culture is not [52].

Consistent with previous research, our study findings demonstrate the differential effects of physical and social disorder on outcome measures [11, 53]. While both types of disorder were found to be directly related to all outcomes, once collective efficacy was added to the model, the relationship between social disorder and each of the outcomes became non-significant. In summary, consistent with the assertions of Sampson and Raudenbush [4] and in concordance with social disorganisation theory, we found that the relationship between social disorder and all outcome measures was significantly mediated by collective efficacy; however, this was not the case for physical disorder. As for the potential reasons for these findings, from a research standpoint, social disorder and physical disorder are qualitatively different: neighbourhood social disorder has been described as “episodic behaviour” involving individuals “which only lasts for a limited amount of time”, whereas neighbourhood physical disorder instead refers to “the deterioration of urban landscapes” and “does not necessarily involve actors” ([53] p5). Similarly, in a hospital setting, physical disorder may be perceived by staff as a more stable and constant presence in the hospital environment. In other words, hospital staff may be “inoculated” ([12] p411) to the presence of physical disorder in the hospital environment, with collective efficacy being less likely to alter or affect the relationship between physical disorder and outcomes.

A further explanation as to why the relationship between social disorder and all three outcome measures were mediated by collective efficacy, but not for physical disorder, is because when social disorder manifests in hospitals (e.g., non-compliance, wasting time), healthcare staff must work together to ‘pick up the slack’ to avoid serious threats to the safety and quality of care delivered. For example, if certain staff are absent or late in a particular hospital ward, the rest of the staff in that ward must work together to negate the likelihood of patient safety issues. Working as a team to make up for the social disorder may prevent any one individual staff member experiencing burnout and low job satisfaction. Indeed, this is consistent with past research showing that collaboration in hospitals has a positive effect on staff and patient outcomes, including patient safety, burnout, and job satisfaction [54]. This differs to physical disorder (e.g., run-down hospital, vandalism) where it is not necessarily seen as the responsibility of hospital staff to work collaboratively and address this form of disorder. That is, while staff must work together to address issues of social disorder such as someone being absent or late, physical disorder is more likely to be seen to be needing to be dealt with on the organisational level. For example, a hospital being in need of repair needs intervention from the government, NHS Trust, Board of Governors or local health district which can provide the necessary resources to redevelop the infrastructure.

This study thereby contributes to the broader BWT and related neighbourhood disorder field as it highlights the importance of keeping social and physical disorder as separate constructs when assessing disorder. Further, this study highlights the importance of encouraging collective efficacy among hospital staff as it can act as a barrier between social disorder and poor staff outcomes and patient safety issues.

### Strengths and limitations

A strength of this study was the development of an initial psychometric profile for the measure of disorder and collective efficacy for hospitals, with its psychometric properties being assessed across four hospital sites in Australia. As to limitations, the study was based on self-reports of staff and, as with all research of this kind, is reflective of the perceptions of the agents involved. We did not include patients’ self-reports or observational research. The data was collected at one time point and therefore cannot identify any causal influence of physical and social disorder on outcomes which would require longitudinal studies involving repeated sampling on the same set of study participants. The findings concerning patient safety would need to be replicated in view of the fact that only one item was used to assess patient safety and therefore the measure has unestablished reliability. The DaCEs also warrants further cross-validation of its factor structure, as the final items were selected on the basis of results from our four included hospitals, and may not be generalisable to all hospital systems. Optimally, CFA should be randomly divided into subgroups (calibration and validation samples) to validate and verify the factor structure of the tool [55]. However, the current study was limited by the relatively modest sample size, and further work would be needed to verify the validity of the tool.

## Conclusions

As one of the first studies to empirically test theories of neighbourhood disorder in healthcare, we found that a positive, orderly, productive culture is likely to lead to wellbeing for staff and better safety for patients, and vice versa. This is a modified study of BWT and related theories in hospitals, and one of the few studies to assess associations between different forms of disorder, collective efficacy, and staff and patient outcomes. Our hypothesised mediation model was supported, showing that the relationship between social disorder and outcomes (job satisfaction, burnout, patient safety) was mediated by collective efficacy. Having established and tested the robustness of the model, we offer it for new applications and future studies on this topic and highlight the importance of studying physical and social disorder as separate constructs. This study demonstrates the potential benefits of encouraging collective efficacy among hospital staff as it can act as a barrier to poor staff wellbeing and patient safety issues when there is social disorder.

## Supplementary Information

**Additional file 1.**

**Additional file 2.**

## Data Availability

The datasets used and/or analysed during the current study are available from the corresponding author on reasonable request.

## Abbreviations

BWT: Broken windows theory
DaCEs: Disorder and Collective Efficacy Survey
CFA: Confirmatory factor analysis
SEM: Structural equation modelling
MBI: Maslach Burnout Inventory
HSOPSC: Hospital Survey of Patient Safety Culture
EM: Expectation Maximisation
TLI: Tucker Lewis Index
CFI: Comparative Fit Index
RMSEA: Root Mean Square Error of Approximation
