# Supplementary material for: An empirical application of “broken windows” and related theories in healthcare: examining disorder, patient safety, staff outcomes, and collective efficacy in hospitals
Source: BMC Health Serv Res. 2020 Dec 4;20:1123. doi: 10.1186/s12913-020-05974-0 (PMC7718712; doi:10.1186/s12913-020-05974-0)
Supplement: Supplementary file 1 — Additional file 1. [file 12913_2020_5974_MOESM1_ESM.docx]

# Supplementary File 1

Descriptive statistics for items assessing Disorder and Collective efficacy

|  | Item | Source | Item | SD | Skewness index | Kurtosis index |
| --- | --- | --- | --- | --- | --- | --- |
| **Disorder** | |  |  |  |  |  |
|  | **Physical disorder** |  |  |  |  |  |
|  | PD1. This hospital is run-down and in disrepair | Perkins and Taylor (1)^a^ | 2.68 | 1.11 | 2.59 | -3.08 |
|  | PD2. There is a lot of broken equipment in this hospital | Plank, Bradshaw (2)^a^ | 2.82 | 1.12 | 1.86 | -2.97 |
|  | PD3. The bathrooms in this hospital are often unclean | Plank, Bradshaw (2)^a^ | 2.98 | 1.15 | 0.94 | -3.71 |
|  | PD4. The interior of this hospital is well kept and in good repair (R) | Researcher-developed | 2.79 | 1.03 | 2.61 | -2.11 |
|  | PD5. The exterior of this hospital is well maintained (R) | Perkins and Taylor (1)^a^ | 2.68 | 1.05 | 3.5 | -1.87 |
|  | PD6. Vandalism of property and equipment is a problem at this hospital | Plank, Bradshaw (2)^a^, Ross and Mirowsky (3)^a^ | 2.34 | 0.88 | 4.16 | 0.88 |
|  | PD7. This hospital is in need of refurbishment | Researcher-developed | 3.39 | 1.23 | -2.73 | -4.05 |
|  | PD8. Broken hospital equipment is fixed in a timely manner (e.g., light bulbs replaced, computer problems resolved) (R) | Researcher-developed | 3.16 | 1.06 | 0.87 | -3.43 |
|  | **Social disorder** | | | | | |
|  | SD1. Using hospital equipment for non-approved purposes | Coyne and Bartram (4)^a^ | 1.94 | 0.79 | 5.80 | 2.64 |
|  | SD2. Absenteeism (i.e., uncertified sick leave or other unauthorised absences) | Coyne and Bartram (4) | 2.38 | 1.05 | 4.37 | -1.19 |
|  | SD3. Lateness (i.e., bad time keeping, general tardiness) | Coyne and Bartram (2000)^a^ | 2.46 | 1.070 | 3.84 | -1.71 |
|  | SD4. Taking breaks without permission | Coyne and Bartram (2000)^a^ | 2.22 | 0.99 | 6.68 | 2.20 |
|  | SD5. Disregard for hospital rules, policies and procedures | Coyne and Bartram (2000)^a^ | 2.10 | 0.96 | 7.98 | 4.65 |
|  | SD6. Wasting time/"slacking" off at work | Coyne and Bartram (2000)^a^ | 2.37 | 1.11 | 5.12 | -0.94 |
|  | SD7. Being under the influence of drugs or alcohol while at work | Coyne and Bartram (2000)^a^ | 1.41 | 0.68 | 15.96 | 24.28 |
|  | SD8. Non-compliance with hospital dress/uniform standards | Researcher-developed | 1.98 | 0.93 | 6.96 | 1.57 |
|  | SD9. Stealing or pilfering items from the ward, other staff or patients | Coyne and Bartram (2000)^a^ | 1.65 | 0.83 | 11.22 | 9.96 |
|  | SD10. Intentional damage to hospital property or equipment by staff or patients | Coyne and Bartram (2000)^a^ | 1.58 | 0.83 | 13.50 | 13.97 |
|  | SD11. Verbal abuse from staff or patients (i.e., communication through words, tone or manner that disparages, patronizes, accuses or is disrespectful) | Plank et al. (2009)^a^, NSW Health (2015)^a^ | 2.76 | 1.32 | 1.05 | -4.65 |
|  | SD12. Violence from staff or patients (i.e., inflicting physical injury, injury, or threat of harm) | Plank et al. (2009)^a^, NSW Health (2015)^a^, Coyne and Bartram (2000)^a^ | 2.40 | 1.29 | 4.40 | -3.29 |
|  | SD13. Discrimination from staff or patients (e.g., on the basis of race, ethnicity, religion) | NSW Health (2015)^a^ | 2.30 | 1.17 | 4.39 | -2.63 |
| **Collective efficacy** | |  |  |  |  |  |
|  | **Social cohesion items** |  |  |  |  |  |
|  | SC1. There is minimal conflict within my unit | Gershon et al. | 3.35 | 1.11 | -4.45 | -2.10 |
|  | SC2. The members of my unit support one another | Gershon et al. | 3.76 | 1.02 | -7.27 | 2.05 |
|  | SC3. On my unit, there is open communication among staff | Gershon et al.^a^ | 3.52 | 1.07 | -4.69 | -1.43 |
|  | SC4. All of the staff in my unit know me by name | Plank et al. (2009)^a^ | 4.12 | .858 | -9.86 | 7.79 |
|  | SC5. Staff in my unit get along well with one another | Plank et al. (2009)^a^ | 3.73 | 0.94 | -7.28 | 3.38 |
|  | SC6. Staff in my unit care about their patients | Plank et al. (2009)^a^ | 4.17 | 0.80 | -9.52 | 10.01 |
|  | SC7. It is easy for staff in this unit to ask questions when there is something that they do not understand | Sexton et al. (2006)^a^ | 3.97 | 0.89 | -9.34 | 7.43 |
|  | SC8. I have the support I need from other staff in my unit to care for patients | Sexton et al. (2006)^a^ | 3.89 | 0.91 | -7.78 | 4.84 |
|  | SC9. Staff in my unit share the same values as me | Researcher-developed | 3.63 | 0.99 | -5.76 | 1.10 |
|  | SC10. The doctors and nurses here work together as a well-coordinated team | Sexton et al. (2006)^a^ | 3.68 | 0.92 | -5.98 | 3.03 |
|  | SC11. In my unit, there is open communication between staff and patients | Researcher-developed | 3.79 | 0.84 | -6.61 | 5.05 |
|  | SC12. In my unit, staff are treated respectfully regardless of their job | NSW Health (2015)^a^ | 3.71 | 1.07 | -7.18 | 1.19 |
|  | **Willingness to intervene** |  |  |  |  |  |
|  | WI1. In my work area, it is difficult to speak up if I perceive a problem with patient care (R) | Sexton et al. (2006)^a^ | 3.61 | 0.99 | -5.55 | 0.59 |
|  | WI2. I know the proper channels to direct questions regarding patient safety | Sexton et al. (2006)^a^ | 3.92 | 0.77 | -7.15 | 6.39 |
|  | WI3. I am encouraged by my colleagues to report any patient safety concerns I may have | Sexton et al. (2006) | 3.83 | 0.82 | -7.16 | 5.44 |
|  | WI4. In my work area, it is difficult to discuss errors (R) | Sexton et al. (2006)^a^ | 3.60 | 1.02 | -6.12 | 0.26 |
|  | WI5. In this unit, it is difficult to speak up if someone is being spoken to rudely or verbally abused by a co-worker or supervisor (R) | NSW Public Service Commission (2016)^a^ | 2.58 | 1.08 | 4.02 | -1.375 |
|  | WI6. My suggestions about patient safety would be acknowledged if I expressed them to senior staff | Sexton et al. (2006)^a^ | 3.73 | 0.90 | -5.32 | 1.72 |
|  | WI7. I know the proper channels to follow if I have a serious complaint to file about unacceptable work behaviours (e.g., bullying, verbal abuse, discrimination) | Sexton et al. (2006)^a^ | 3.93 | 0.84 | -9.48 | 9.30 |
|  | WI8. In this unit, it is safe to speak up and challenge the way things are done | NSW Health (2015)^a^ | 3.397 | 1.08 | -5.20 | -0.85 |
|  | WI9. I have confidence that if I were to report unacceptable work behaviours (e.g., bullying, verbal abuse, discrimination) that they would be responded to appropriately) | NSW Health (2015)^a^ | 3.26 | 1.12 | -4.36 | -2.11 |
|  | WI10. The culture in my work area makes it easy to learn from the errors of others | Sexton et al. (2006)^a^ | 3.48 | 0.96 | -4.92 | 0.49 |

(R) Items were reverse coded, ^a^ Adapted for the hospital context

**References**

Coyne I, Bartram D. Personnel managers' perceptions of dishonesty in the workplace. *Hum Resour Manag J*. 2000;10:38.

Gershon RRM, Karkashian CD, Grosch JW, Murphy LR, Escamilla-Cejudo A, Flanagan PA, et al. Hospital safety climate and its relationship with safe work practices and workplace exposure incidents. *American Journal of Infection Control*. 2000;28:211-21.

| NSW Health. 2015 YourSay Workplace Culture Survey. <https://www.health.nsw.gov.au/workforce/yoursay/2015/Pages/default.aspx>. |
| --- |

NSW Public Service Commission. 2016 People Matter Employee Survey. <https://www.psc.nsw.gov.au/reports---data/people-matter-employee-survey/previous-surveys/people-matter-employee-survey-2016>.

Plank Stephen B, Bradshaw Catherine P, Young H. An application of “Broken‐Windows” and related theories to the study of disorder, fear, and collective efficacy in schools. *American Journal of Education*. 2009;115:227-47.

Perkins DD, Taylor RB. Ecological Assessments of Community Disorder: Their Relationship to Fear of Crime and Theoretical Implications. In: Revenson TA, D’Augelli AR, French SE, Hughes DL, Livert D, Seidman E, et al., editors. Ecological Research to Promote Social Change: Methodological Advances from Community Psychology. Boston, MA: Springer US; 2002. p. 127-70.

Ross CE, Mirowsky J. Disorder and decay: The concept and measurement of perceived neighborhood disorder. *Urban Affairs Review*. 1999;34:412-32.

Sexton JB, Helmreich RL, Neilands TB, Rowan K, Vella K, Boyden J, Roberts PR, Thomas EJ. The Safety Attitudes Questionnaire: psychometric properties, benchmarking data, and emerging research. BMC health services research. 2006 Dec 1;6(1):44.
