# Supplementary material for: An empirical application of “broken windows” and related theories in healthcare: examining disorder, patient safety, staff outcomes, and collective efficacy in hospitals
Source: BMC Health Serv Res. 2020 Dec 4;20:1123. doi: 10.1186/s12913-020-05974-0 (PMC7718712; doi:10.1186/s12913-020-05974-0)
Supplement: Supplementary file 2 — Additional file 2. [file 12913_2020_5974_MOESM2_ESM.docx]

# Supplementary File 2

Summary of Goodness of Fit for direct effect models

| Model | x^2^ | df | CFI | TLI | RMSEA |
| --- | --- | --- | --- | --- | --- |
| Burnout | 29.548 | 12 | .982 | .968 | .066 |
| Job satisfaction | 98.263 | 32 | .962 | .946 | .078 |
| Patient safety | 45.990 | 12 | .965 | .939 | .091 |
